# Molecular Studies in *Treponema pallidum* Evolution: Toward Clarity?

**DOI:** 10.1371/journal.pntd.0000184

**Published:** 2008-01-23

**Authors:** Connie J. Mulligan, Steven J. Norris, Sheila A. Lukehart

**Affiliations:** 1 Department of Anthropology, University of Florida, Gainesville, Florida, United States of America; 2 Department of Pathology and Laboratory Medicine, University of Texas-Houston Medical School, Houston, Texas, United States of America; 3 Department of Medicine, University of Washington, Seattle, Washington, United States of America; Fundação Oswaldo Cruz, Brazil

## Background

Syphilis is the best known treponemal infection and the disease has captured the attention of well-known physicians and the imaginations of writers and artists. In contrast, the nonvenereal treponemal infections (yaws, bejel, and pinta) have been endemic in remote regions of Africa, Southeast Asia, and South America, and are thus less well-known in western culture. However, these endemic treponematoses were so prevalent that, between 1952 and 1964, the World Health Organization (WHO) undertook a massive eradication campaign in which over 300 million people in Africa, South America, Southeast Asia, the South Pacific islands, and the Middle East were examined and ∼50 million were treated with penicillin [1]. It is estimated that the burden of disease was reduced by 95%. In the mid 1980s, the Fogarty International Center and WHO sponsored a series of regional meetings to gather information about the status of the endemic treponematoses [2]–[4]. Numerous foci of infection were identified in Africa, Asia, and South America, and the recommendation was made to focus new efforts on control, with the goal of elimination. The HIV epidemic intervened and the attention of the global health community was appropriately diverted to AIDS, thus interrupting the momentum for elimination of the treponematoses. Since that time, because of little to no surveillance in many areas, the current magnitude of the diseases is unknown, although foci of yaws and endemic syphilis have periodically been reported. Several countries, including Indonesia and India, have current ongoing yaws control programs. In 2007, the WHO once again committed itself to eliminate yaws (http://www.who.int/mediacentre/news/notes/2007/np04/en/index.html).

Traditionally, the human Treponematoses have been differentiated based upon their mode of transmission (sexual vs. non-venereal), clinical manifestations, serious sequelae (e.g. central nervous system [CNS] or cardiovascular involvement, congenital infection), and experimental host range (Table 1). In the mid-1900's, a disagreement raged over whether the etiologic agents of these infections were the same species, with mode of transmission and clinical course defined by culture and climate, or were different species with important biological differences [5],[6]. The agents are morphologically identical, and all induce reactivity in the standard serological tests used for syphilis diagnosis. Recent molecular studies have identified genetic signatures that can differentiate the existing strains of *Treponema pallidum* subspecies *pallidum* (syphilis), subspecies *pertenue* (yaws), and subspecies *endemicum* (bejel) [7], yet the molecular bases for the described differences in transmission and clinical course have not been defined.

**Table 1:** Traditional Characteristics of Treponemal Infections

Species	Disease	Mode of Transmission	Serious Sequelae	Preferred Experimental Host
*T. pallidum* subsp. *pallidum*	Syphilis	Sexual	Cardiovascular and CNS disease, gummas, congenital syphilis	Rabbit
*T. pallidum* subsp. *pertenue*	Yaws	Direct nonsexual contact	Gummas	Hamster
*T. pallidum* subsp. *endemicum*	Bejel	Direct nonsexual contact	Gummas	Hamster
*T. carateum*	Pinta	Direct nonsexual contact	None	Non-human primate

How definitive are these clinical and epidemiological differences? Syphilis is usually transmitted sexually, but there are many examples in older textbooks of nonsexual transmission via direct contact; examples include digital infection of dentists via contact with oral lesions of syphilis patients, and infection of the nipple in wet nurses via oral lesions in infected infants. In contrast, yaws is generally transmitted by skin-to-skin contact in children, and infectious lesions are typically resolved before sexual debut, thus transmission during sexual activities is not recognized. Thus, mode of transmission appears to be defined by opportunity, rather than biology. There is similar overlap in clinical manifestations. The lesions of yaws are typically described as “frambesiform” (raised “berry-like” lesions compared to the ulcer or maculopapular rash of syphilis) yet there are numerous descriptions in the literature of less dramatic, even macular, yaws lesions in drier climates [8]. Conversely, the condylomata lata of secondary syphilis can be raised and multilobed (“frambesiform”), and they appear in moist body folds, a common location for the frambesiform lesions of secondary yaws. Although gummatous destruction, particularly of bone and cartilage, is well-recognized in syphilis, yaws, and bejel, only syphilis is said to lead to CNS involvement and congenital infection. However, in a very thoroughly researched review article, Roman and Roman [9] argue eloquently that there is ample evidence for CNS, cardiovascular, and congenital infection in yaws. In that review, Blacklock [10] is quoted as stating that the “criteria used to differentiate syphilis and yaws were ‘nothing more than the statement of an epidemiological observation.’” As an example, the Haiti B strain was originally identified as *T. pallidum* subsp. *pertenue* because it was isolated from an 11-year old child with “typical generalized frambesiform yaws” [11], in a epidemiological setting of childhood infection. Yet molecular studies in several labs [12]–[14] have subsequently demonstrated that the Haiti B strain has molecular signatures consistent with *T. pallidum* subsp. *pallidum*. The Madras strain is another such example of a “yaws” isolate that appears in fact to be a *pallidum* strain based on molecular data. With regard to host range differences, the Haiti B strain was used extensively by Schell et al. [15]–[17] in their studies of yaws infection in the hamster model. It has been stated that hamsters do not regularly develop clinical manifestations of syphilitic disease [11],[17], yet hamsters are readily infected by the Haiti B strain, now considered to be a *pallidum* strain. Thus the clinical, epidemiological, and host range criteria used to differentiate the agents of the treponematoses are soft.

Molecular studies have identified signature polymorphisms that serve to differentiate the known strains of the three subspecies *pallidum*, *pertenue*, and *endemicum* [7]. While these studies suggest that there are genetic differences among subspecies, the strains used to identify these distinctive signatures were classified by the clinical and epidemiological criteria described above and thus the molecular signatures may be biased. As mentioned above, two strains (Haiti B and Madras) were isolated from characteristic yaws lesions, yet they have the molecular signatures of the *pallidum* subspecies.

## A New Study: On the Origin of the Treponematoses—A Phylogenetic Approach

Harper and colleagues [18] have examined a number of chromosomal regions in a total of 23 strains/samples representing the three *T. pallidum* subspecies and have identified regions of mutation (single nucleotide polymorphisms [SNP] or indels). Some of these polymorphisms have been previously described and others are novel. Importantly, this analysis included two new yaws samples from Guyana, representing the only South American yaws samples evaluated to date. Based upon 17 regions of mutation in the established strains, the authors constructed a phylogenetic tree of the concatenated sequences, and propose that this tree identifies the yaws subspecies as being the oldest, with the bejel and syphilis subspecies evolving subsequently. Unfortunately the DNA from the two new yaws samples from Guyana was too degraded to conduct extensive sequencing, so these samples were not included in the phylogenetic analysis. From these samples, the authors selected a subset of seven genetic regions for analysis. Based upon the homology of four SNPs in the Guyana samples with the group of *pallidum* strains, the authors conclude that the syphilis subspecies evolved from New World yaws strains.

## Strengths and Limitations of the Study

The basis of the claim for a New World origin of venereal syphilis is sequence similarity between the Guyana yaws samples and the *pallidum* strains. However, the sequence similarity is based on only four SNPs. Furthermore, three of the SNPs cause non-synonymous changes and occur in a very short region (∼15 amino acids) of the *tprI* protein. This is an extraordinarily high rate of evolutionary change in a genus that has been characterized by very little change. Two of the SNPs (*tprI* 137 and 151) are shown to have evolved two independent times according to the authors' network analysis (Fig. 3 of Harper et al. [18]), a result that again makes little sense in a genus characterized by very little variation. One of those SNPs (*tprI* 151) also differs between two *pertenue* strains CDC-1 and CDC-2, which are strains that might be predicted to be identical given the very close geographical and chronological proximity of isolation. Finally, *tprI* is thought to be involved in pathogenesis [19] and thus is subject to the effects of natural selection, which violates the assumptions of phylogenetic analysis. Clearly the *tprI* locus is atypical of the treponemal genome and, thus, is not the best choice when trying to resolve the decades-old debate concerning the origin of venereal syphilis.

**Figure 1:**
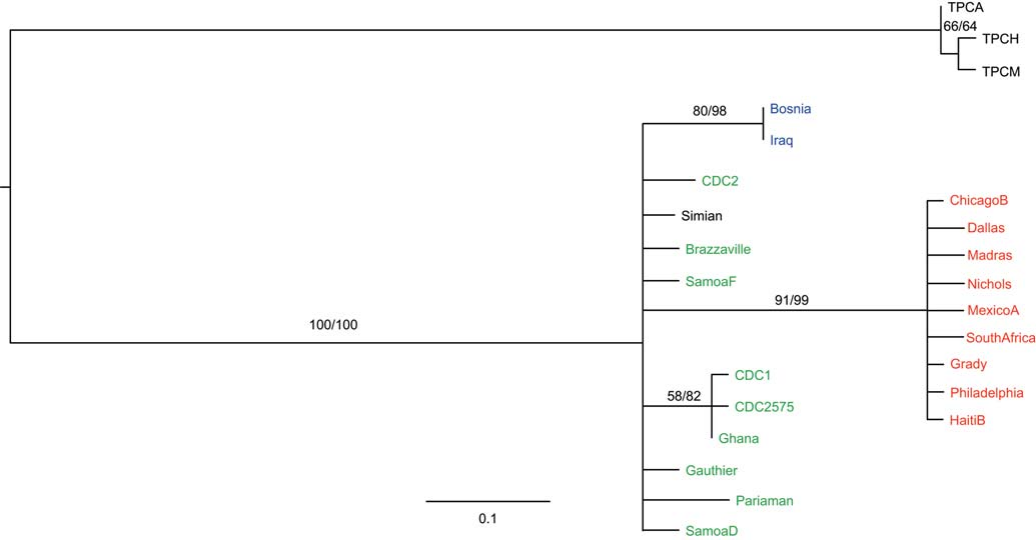
A Phylogenetic Tree Depicting the Relationships between *T. pallidum* strains. This phylogeny is identical to the phylogeny of Harper et al. (Fig. 2) except that it has been re-estimated to show only branches with >50% bootstrap support for both maximum likelihood and maximum parsimony analyses. As with Harper et al.'s phylogeny, vertical distance is not meaningful, i.e. the placement of taxa along the vertical lines is inconsequential. *Pertenue*, *endemicum* and *pallidum* strains are depicted in green, blue, and red, respectively.

Additionally, the phylogenetic and network analyses presented by Harper et al. are contradictory in that the phylogeny supposedly supports the evolution of *pallidum* from *endemicum* (Fig 2 of Harper et al. [18]) but the network (Fig 3 of Harper et al. [18]) is used to infer the origin of *pallidum* from New World *pertenue* strains. Part of the problem may be the fact that the phylogeny does not show significant structure, contrary to the authors' claims. When the tree is redrawn to show only branches with minimal 50% bootstrap support, the *pertenue* cluster disappears and all three subspecies, plus the simian isolate, branch off the most basal branch simultaneously (see Figure 1). This means that no evolutionary order can be inferred. Furthermore, since all strains were collected contemporaneously (at least on an evolutionary time scale), the branch lengths should all be approximately equivalent since a phylogeny reflects only mutational evolution (i.e. all treponemal strains should be equidistant from their common ancestor). The fact that the *pallidum* strains have longer branch lengths does not mean they evolved more recently, but instead is consistent with an argument for increased recombination or selection along the *pallidum* branch, i.e. essentially any phenomenon that violates the evolution-by-mutation-only assumption of a phylogenetic analysis. It is also perhaps noteworthy that the *pallidum* strains are all from the New World except for two strains (South Africa and Madras), whereas the *endemicum* and *pertenue* strains are all from the Old World (with the exception of the new Guyana strains). Thus, the reported sequence homology between the Guyana and *pallidum* strains may simply reflect geographic clustering of New World vs. Old World strains. It would be interesting, though perhaps not possible, to examine older European or Asian *pallidum* strains to see whether the phylogeny is altered.

Caution, therefore, must be used in drawing conclusions about the evolution of “subspecies” that may represent a biological continuum, rather than discrete agents. Certainly, firm conclusions should not be based upon a few SNPs in two samples taken from a single location.

## Next Steps

Despite the limitations of the Harper et al. analysis (as acknowledged by the authors in the discussion), the results reinforce another long-term question: How could the limited divergence between *Treponema* species and subspecies give rise to the observed differences in pathogenesis? Examination of ∼7 kb of sequence resulted in the identification of only 26 nucleotide substitutions among *T. pallidum* strains, or one difference for every 275 bp (99.6% identity). As indicated in the article, this figure most likely represents a gross overestimate of the overall degree of heterogeneity, because some of the DNA segments were selected because of their high variability. A recent report by Strouhal et al. [20] compared the sequences of *T. pallidum* subsp. *pallidum* (Nichols) and *Treponema paraluiscuniculi* (Strain Cuniculi A), the closely related spirochete that causes venereal spirochaetosis in rabbits but is not pathogenic to humans. A genome-wide analysis using microarray and whole genome restriction mapping indicated that the overall sequence similarity is in the range of 98.6 to 99.3%. Most of the differences identified are within *tpr* genes or neighboring genes. Based on these two studies and prior evidence, it is possible that genes within the *tpr* loci are primarily responsible for the differences in disease manifestations and host susceptibility.

*T. pallidum* is one of the few human bacterial pathogens that have not been cultivated in vitro, obviating experimental approaches such as mutational analysis and complementation to definitively identify virulence determinants. How then can our knowledge of genes related to treponemal evolution and pathogenesis be further refined? One possible approach is whole genome sequencing of multiple strains and comparison of the resulting sequences. The ongoing goal of inexpensive, ‘personalized’ human genome sequences has resulted in the development of multiple novel sequencing approaches; some of these methods can yield a high redundancy bacterial genome sequence for ∼US$400 in reagent costs [21]. The new methodologies tend to yield shorter sequences per template (25 to 100 bp) and to have a higher error rate than Sanger sequencing. These shortcomings make the newer technologies more applicable to genome re-sequencing (e.g. the analysis of the closely related pathogenic *Treponema* strains) and can be ameliorated in part by combining the results of two or more sequencing technologies.

Because of the paucity of available *endemicum* strains and New World *pertenue* isolates, new approaches may be needed to analyze archival DNA specimens. Kolman et al. [22] were able to identify *T. pallidum* sequences by PCR amplification of DNA extracted from deformed bones (saber shins) in 200-year-old skeletal remains from Easter Island. Processes have been developed for isolation, whole genome amplification, and sequencing of DNA from individual bacterial cells [23],[24]. These methods, perhaps coupled with laser capture microscopy or techniques for dissecting out organisms, could be utilized to recover and obtain sequence information from ancient bones or preserved tissue specimens with treponemal infections.
